# AKT Isoforms Interplay in High-Grade Serous Ovarian Cancer Prognosis and Characterization

**DOI:** 10.3390/cancers14020304

**Published:** 2022-01-08

**Authors:** Eros Azzalini, Domenico Tierno, Michele Bartoletti, Renzo Barbazza, Giorgio Giorda, Fabio Puglisi, Sabrina Chiara Cecere, Nunzia Simona Losito, Daniela Russo, Giorgio Stanta, Vincenzo Canzonieri, Serena Bonin

**Affiliations:** 1 Department of Medical Sciences (DSM), University of Trieste, 34147 Trieste, Italy; eazzalini@units.it (E.A.); domenico.tierno@phd.units.it (D.T.); renzo51@gmail.com (R.B.); stanta@units.it (G.S.); 2 Pathology Unit, IRCCS CRO Aviano-National Cancer Institute, 33081 Aviano, Italy; 3 Unit of Medical Oncology and Cancer Prevention, Department of Medical Oncology, IRCCS CRO Aviano-National Cancer Institute, 33081 Aviano, Italy; michele.bartoletti@cro.it (M.B.); fabio.puglisi@cro.it (F.P.); 4 Department of Medicine (DAME), University of Udine, 33100 Udine, Italy; 5 Unit of Gynecologic Oncology Surgery, IRCCS CRO Aviano, National Cancer Institute, 33081 Aviano, Italy; ggiorda@cro.it; 6 Istituto Nazionale Tumori IRCCS—Fondazione G. Pascale, 80131 Napoli, Italy; s.cecere@istitutotumori.na.it (S.C.C.); n.losito@istitutotumori.na.it (N.S.L.); d.russo@istitutotumori.na.it (D.R.)

**Keywords:** HGSOC, AKT1, AKT2, AKT3, SET, classic, surrogate marker, BRCA status, homologous recombination status

## Abstract

**Simple Summary:**

New therapeutical strategies are needed to improve survival in high-grade serous ovarian cancer (HGSOC) patients. AKT inhibitors are promising agents able to act in synergy with PARP inhibitors and platinum-based therapies, but the subset of patients who could benefit from this approach is still unclear. We analyzed AKT isoforms expression in a retrospective cohort and we identified four AKT expression groups related to patients’ survival, tumor morphology and the BRCA status that could help in stratifying patients for future clinical trials.

**Abstract:**

High-grade serous ovarian cancer (HGSOC) is among the deadliest gynecological malignancies. The acquired resistance to platinum-based therapies and the intrinsic heterogeneity of the disease contribute to the low survival rate. To improve patients’ outcomes, new combinatorial approaches able to target different tumor vulnerabilities and enhance the efficacy of the current therapies are required. AKT inhibitors are promising antineoplastic agents able to act in synergy with PARP inhibitors, but the spectrum of patients who can benefit from this combination is unclear, since the role of the three different isoforms of AKT is still unknown. Here, we study the expression of AKT isoforms on a retrospective cohort of archive tissue by RT-droplet digital PCR (ddPCR) analyzing their association with the clinicopathological features of patients. Based on AKT1/AKT2 and AKT1/AKT3 ratios, we define four AKT classes which were related to patients’ survival, tumor morphology and BRCA1 expression. Moreover, our results show that high AKT3 expression levels were frequently associated with tumors having classic features, a low number of mitoses and the presence of psammoma bodies. Overall, our study obtains new insights on AKT isoforms and their associations with the clinicopathological features of HGSOC patients. These evidences could help to better define the subsets of patients who can benefit from AKT and PARP inhibitors therapy in future clinical trials.

## 1. Introduction

High-grade serous ovarian cancer (HGSOC) accounts for around 70% of epithelial ovarian carcinomas (EOC) and it is among the deadliest gynecological malignancies. There is an uncertainty on the cells of origin for HGSOC, which has been reported to arise both from the inner surface of fallopian tubes and from the ovarian surface epithelium [1]. HGSOC patients are typically diagnosed at a median age of 60 years with a late-stage disease often asymptomatic. The survival rate in the advanced stages is very poor, since only 30% of women survive after five years from diagnosis [2]. This high mortality rate is due to the lack of early detection and the acquired resistance to current chemotherapies, mostly platinum and taxane-based as first-line agents [3].

In recent years, the introduction of PARPs inhibitors as a therapeutical strategy for HGSOC has significantly improved patients’ prognosis, especially in women harboring BRCA1 and BRCA2 mutations [4]. However, given the high heterogeneity of the disease, most patients develop PARP resistance and cancer progression [5]. Therefore, there’s an urgent need for developing combinatorial and personalized approaches able to contrast the tumor on different fronts and increase the efficacy of both first-line agents and PARPs inhibitors.

In connection therewith, several efforts have been undertaken in studying the effect of the PI3K/AKT pathway on BRCA1/2 expression and the synergistic activity of PI3K/AKT inhibitors with PARPs inhibitors [6,7].

The signaling cascades induced by PI3K activation lead to cell growth, proliferation and survival and, therefore, have an important role in tumor progression [8]. Moreover, since the PI3K/AKT pathway is altered in almost 45% of serous ovarian carcinomas [9], it represents an attractive therapeutical point.

Among the molecules involved in PI3K signaling, AKT is an interesting target, since it regulates several downstream effectors and cellular functions [10]. In humans, AKT has three different isoforms encoded by three different genes, namely, AKT1, AKT2 and AKT3. Despite the high homology of the three isoforms, they have been reported to play different or even opposite roles in a variety of cancers [11,12], with concerns on the therapeutic approach. AKT isoforms can specifically regulate several cellular processes. While all isoforms seem to downregulate autophagy, AKT1 and AKT3 seem to be involved in cell proliferation and AKT1 and AKT2 in the regulation of the cancer cell metabolism [13].

In HGSOC, several studies on cell lines have shown that the treatment with pan-AKT inhibitors can be effective only in a subset of tumors, while in others, an isoform-specific approach should be preferred [14]. Moreover, a recent meta-analysis on the use of monotherapy with PI3K/AKT/mTOR pathway inhibitors in ovarian cancers confirmed their limited benefit in clinic. However, the main biases in these clinical trials were the prevalent use of pan-AKT drugs rather than the isoform-specific ones and the high therapy toxicity [15].

Taking that into consideration, there’s a general consensus in favor that only a subset of patients can benefit from AKT inhibitors, but their characterization is still unclear.

In this study, we analyze the expression of AKT1, AKT2 and AKT3 isoforms in a retrospective cohort of archive HGSOC tissues to evaluate their role in patients’ prognosis. Furthermore, the possible associations with tumor morphology and BRCA1 expression have also been investigated to better define patients that could benefit from combinatorial therapies with PARPs and AKT inhibitors.

## 2. Materials and Methods

### 2.1. Cases Selection

A total of 121 high-grade serous ovarian carcinomas diagnosed from 1999 to 2019 were included in the study. The hematoxylin and eosin-stained slides of 100 samples together with the respective paraffin blocks were retrieved from the archives of the National Cancer Institute of Aviano (CRO), while 21 samples from the National Cancer Institute “G. Pascale” of Naples. Samples from CRO have already been characterized in two previous studies [16,17].

All patients gave informed consent before enrollment in the study that was conducted in accordance with the Declaration of Helsinki. The study was approved by the institutional review board (protocol number 1213, 24 January 2017).

Inclusion criteria were patients with (i) advanced stage disease (FIGO III-IV) who were not treated with neoadjuvant chemotherapy (NACT), (ii) partial or complete clinical information available, (iii) H&E slides and respective paraffin blocks available. Data on demographics and clinical follow up were obtained from medical records and hospitals’ databases.

### 2.2. Histological Review

H&E sections were reviewed by expert pathologists (R.B., G.S., N.S.L. and V.C.) to determine the histo-morphological features according to Soslow and colleagues [18]. The assessment of the number of mitoses and lymphocytes, tumor growth patterns and SET/Classic groups was carried out as previously described [17].

### 2.3. RNA Extraction and Reverse Transcription

One 10 µm thick tissue section was cut for each tissue block and total RNA was isolated using the Maxwell^®^ RSC RNA FFPE kit (Cat. no. 1440, Promega, Madison, WI 53711-5399, USA) on the dedicated automated system as described elsewhere [19]. RNA isolation was performed following the manufacturer’s instructions except for the digestion step with proteinase K that was extended to overnight. Total RNA was eluted in 50 µL of RNAse free water, aliquoted and stored at −80 °C.

Next, for each sample, 300 ng of total RNA was reverse transcribed using random primers and 200 U/µL M-MLV reverse transcriptase as previously described [20]. Complementary DNA was split into aliquots and stored at −80 °C until use.

### 2.4. Droplet Digital PCR

The expression levels of AKT isoforms (AKT1, AKT2, AKT3) and two housekeeping genes used for normalization (ACTB and HPRT1) were detected by droplet digital PCR. The reaction was carried out in 20 µL final volume containing 1X ddPCR™ Supermix for Probes (no dUTP) (Biorad, Hercules, CA, USA; Cat. No. 1863024), 17 ng of cDNA, 18 pmol of each primer and 5 pmol of the specific probe. As negative control, a no-template sample containing RNAse-free water was included in each run. Droplets were generated in the QX200™ droplet generator (BioRad, Hercules, CA 94547, USA), transferred to a dedicated PCR 96-well plate (BioRad, Hercules, CA 94547, USA) and then sealed two times at 176 °C. The cDNA included in the droplet emulsion was then amplified on a thermocycler (iCycler, BioRad, Hercules, CA, USA) following the manufacturer’s instructions with a slight modification of the ramp rate which was slowed down to 1 °C/sec. The proper annealing temperature for each amplicon was determined by thermal gradient. Primers and probe sequences were already reported [21] except for AKT1 (see Table A1 for details).

Eventually, the fluorescence amplitude of each droplet was detected by the Droplet Reader™ instrument (BioRad, Hercules, CA, USA) and expressed as absolute counts (copies/µL).

### 2.5. BRCA1 Immunohistochemistry and Homologous Recombination (HR) Status

The BRCA1 immunostaining was performed on whole tissue specimens using the automated XT iVIEW DAB v.1 protocol (Ventana-Roche Diagnostics, Tucson, AZ 85755, USA) on the Benchmark ULTRA IHC/ISH Staining Module (Ventana-Roche Diagnostics, Tucson, AZ 85755, USA). Slides were incubated with BRCA1 antibody (Abcam, Cambridge, UK; Cat. No. ab16780) for 36 min at room temperature and the signal was detected with I-View DAB detection system. The semi-quantitative analysis of the antibody staining was performed using the H-score method as described elsewhere [17].

Homologous recombination (HR) status by BRCA1/BRCA2 genetic testing was available for 36 patients. Women with somatic pathogenic mutations on BRCA1 or BRCA2 genes were considered HR-deficient (HRD), while those without pathogenic variants HR-proficient (HRP).

### 2.6. Data validation

Results on the association between AKT isoform categories and BRCA1 expression were validated using a public available ovarian cancer dataset (Ovarian Serous Cystadenocarcinoma—TCGA Firehose Legacy). Clinical information and gene expression profiles (Affymetrix microarray) were downloaded from http://www.cbioportal.org (accessed on 4 October 2021). Patients with FIGO stage I–II, as well as tumor samples obtained from ascitic fluid and fine needle aspiration, were excluded. The normalized expression values for AKT isoforms and BRCA1 were calculated as previously described in Section 2.4.

### 2.7. Statistical Analysis

Droplet digital PCR expression values for AKT isoforms were normalized using ACTB and HPRT1 genes according to the method described in gene expression data analysis guidelines of nSolver software (https://www.nanostring.com) (accessed on 22 December 2021). Samples with poor cDNA quality having normalization factors higher than 6 were discarded. The comparison between continuous variables and AKT patterns was evaluated using Kruskal–Wallis test, while Pearson’s chi-square test was used for categorical variables. Variables’ correlation was estimated by Spearman’s rank test.

Overall survival (OS) was defined as the time from HGSOC diagnosis to end of follow-up information or death, whichever came first. Progression-free survival (PFS) was established as the time between the start of first-line chemotherapy and disease progression or death. Patients’ response to first-line agents was divided into three groups according to the platinum-free interval (PFI) length. Women with no progression after primary therapy were classified as “never progressed”, those with a PFI < 6 months as “resistant” and those with PFI > 6 months as “sensitive”.

For survival analysis, AKT gene expression was dichotomized according to the median value in “high” and “low” groups. The prognostic significance of the variables was determined by log-rank test and Cox regression analysis. In the case of multivariate Cox regression, the proportional hazard assumption was checked by Shoenfeld’s residuals method. A *p*-value < 0.05 was considered statistically significant.

Statistical analyses were performed using R software (ver. 4.0.2, R Foundation for Statistical Computing, Vienna, Austria) and GraphPad Prism 8 (La Jolla, CA, USA).

## 3. Results

### 3.1. Patients’ Features

Among the 121 HGSOC samples included in the study, eighteen were discarded due to a poor RNA quality. Clinicopathological features of the remaining 103 women enrolled are reported in Table 1. The median overall survival (OS) was 41 months, while progression-free survival (PFS) was 14 months. The survival status was missing in one patient. On average, the 5-year survival rate was 32%.

After a histological revision, the following HGSOC growth patterns were detected: 20 infiltrative, 2 micropapillary, 31 papillary, 24 pseudo-endometrioid, 18 solid and 8 transitional-like. A total of 57 tumors had prevalent SET features, while 43 had prevalent classic features.

### 3.2. AKT Isoforms Are Predictive of Patients’ Survival

Median number of copies/µL for AKT1, AKT2 and AKT3 were, respectively, 98, 61 and 17. In most patients, AKT1 was the prevalent isoform (68%), followed by AKT2 (29%) and AKT3 (3%).

**Figure 1:**
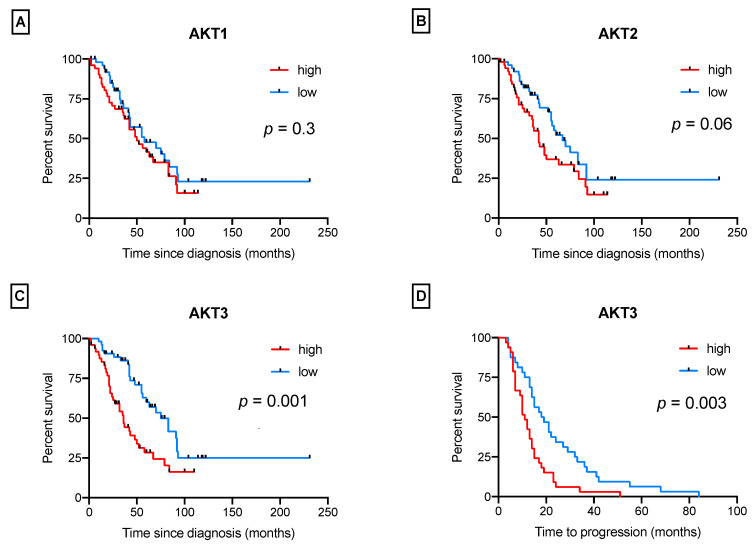
Kaplan–Meier survival curves for AKT isoforms expression, notably, overall survival for AKT1 (**A**), AKT2 (**B**) and AKT3 (**C**); (**D**) progression-free survival (PFS) for p AKT3 expression.

Regarding the influence of AKTs isoforms on patients’ survival, high AKT3 expression levels were significantly related to a shorter overall survival (HR = 2.31; CI 1.38–3.88; *p* = 0.001), while AKT2 and AKT1 had, respectively, a borderline association (HR = 1.61; CI 0.97–2.70; *p* = 0.06) and no association (HR = 1.32; CI 0.79–2.21; *p* = 0.3) (Figure 1A–C). Similarly, high expression levels of AKT1 and AKT2 did not affect progression-free survival (*p* = 0.7 and 0.09, respectively), while AKT3 was significantly associated with an earlier relapse (HR 2.19; CI 1.30–3.66; *p* = 0.003) (Figure 1D).

**Figure A1:**
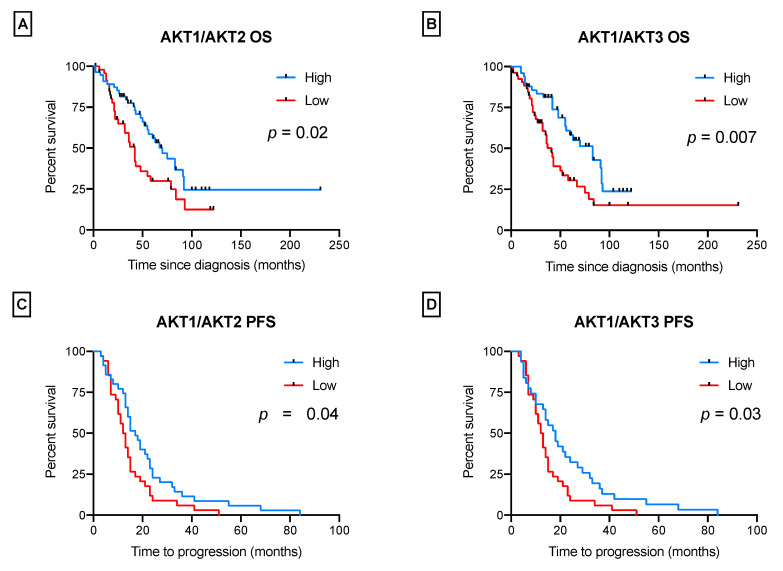
Kaplan–Meier survival curves for AKT isoform expression ratios. Overall survival for AKT1/AKT2 (**A**) and AKT1/AKT3 (**B**) ratios; progression-free survival (PFS) for AKT1/AKT2 (**C**) and AKT1/AKT3 (**D**) ratios. OS—overall survival; PFS—progression-free survival.

According to these results, AKT3 seemed to predict patients’ outcome. Nevertheless, to test the reciprocal expression of the three AKT genes in determining prognosis, we calculated the ratios between the AKT1 normalized expression level and those of AKT2 and AKT3 isoforms. For each ratio, values were dichotomized into “high” and “low” groups, according to the median value (1.6 for AKT1/AKT2 and 5.8 for AKT1/AKT3). “High” groups were indicative of prevalent AKT1 expression over the other two isoforms, while the opposite for “low” groups. The log-rank test returned that low values of AKT1/AKT2 and AKT1/AKT3 were both indicative of a shorter overall survival (*p* = 0.02 and *p* = 0.007, respectively), as well as a shorter time to recurrence (*p* = 0.04 and *p* = 0.03, respectively) as depicted in Figure A1.

By combining the “high” and “low” groups of the two AKT ratios, four categories were obtained as follows:

“high/high”: AKT1 was prevalent over AKT2 and AKT3 (ratios ≥ 1.6 and ≥ 5.8);
“high/low”: AKT1 was over AKT2, but not over AKT3 (ratios ≥ 1.6 and < 5.8);
“low/high”: AKT1 was over AKT3, but not over AKT2 (ratios < 1.6 and ≥ 5.8);
“low/low”: AKT1 was not prevalent over any isoform (AKT2 and AKT3) (ratios < 1.6 and < 5.8).

**Figure 2:**
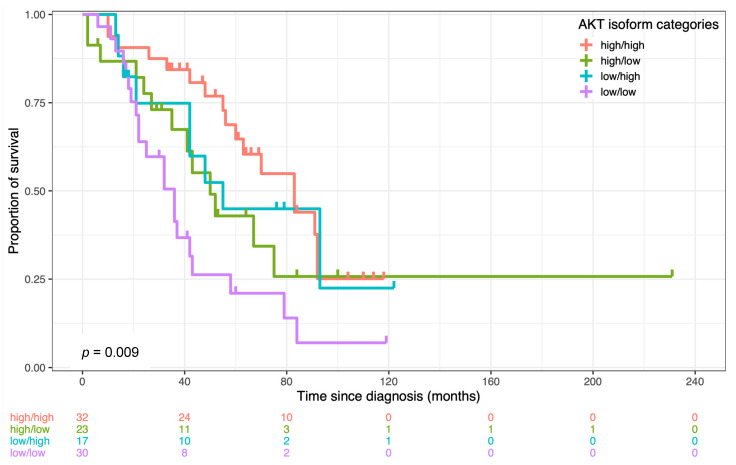
Kaplan–Meier curves representing the overall survival rate by AKT isoform categories.

The abovementioned categories influenced both OS and PFS. In detail, AKT groups were strongly associated with patients’ overall survival (*p* = 0.009) (Figure 2) and moderately with progression-free survival (*p* = 0.06). The univariate Cox-regression analysis showed that “low/low” patients, where both AKT2 and AKT3 prevailed over AKT1, had a risk of death and recurrence that was, respectively, three (HR = 2.92; CI 1.52–5.6; *p* = 0.001) and two (HR = 2.40; CI 1.26–4.65; *p* = 0.008) times higher than women included in the “high/high” group (prevalence of AKT1 isoform). The other two groups (“high/low” and “low/high”), instead, had intermediate overall and progression-free survivals.

According to the completeness of clinical information, a Cox multivariate regression analysis was performed for 93 patients, including as the covariates age at diagnosis, FIGO stage and the residual tumor after surgery. Results confirmed AKT categories as independent prognostic factors for OS in HGSOC patients (Table 2). Data for PFS are reported in Appendix A Table A2.

### 3.3. AKT Isoform Ratios Are Related to HGSOC Morphology and Proliferation

Next, the four classes were related to patients’ histological and clinical features. AKT isoform categories did not correlate with the amount of lymphocytic infiltrate, the residual tumor after surgery, the FIGO stage and the age at diagnosis, but they varied significantly with respect to the architectural growth patterns as defined by Soslow and colleagues [18], mitoses and the presence of psammoma bodies as shown in Table 3.

**Figure 3:**
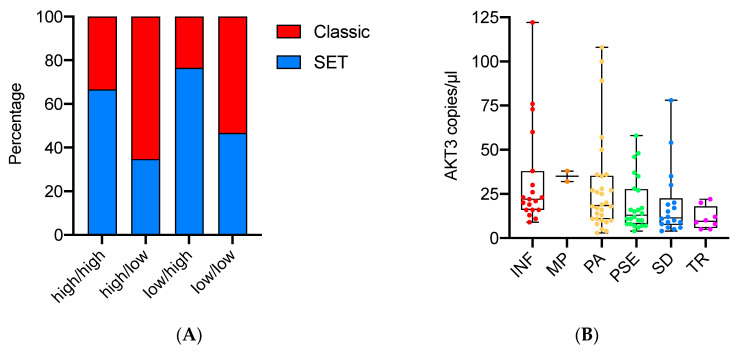
Association between AKT isoform expression categories and tumor morphology. (**A**) Bar chart plot showing the distribution of SET (solid, pseudo-endometrioid, transitional) and classic features among the four AKT classes; (**B**) box plots representing AKT3 expression among the six HGSOC growth patterns. INF—infiltrative; MP—micropapillary; PA—papillary; PSE—pseudo-endometrioid; SD—solid; TR—transitional-like.

The four classes were differentially distributed among tumors with SET/Classic features (*p* = 0.02) (Figure 3A) and among HGSOC growth patterns (*p* = 0.01). Additionally, groups with prevalent AKT3 expression (“high/low” and “low/low”) had the lowest number of mitoses (*p* = 0.03) and the highest frequency of psammoma bodies (*p* = 0.03).

AKT3 was, indeed, a major determinant of tumor architecture, since its expression (copies/µL) was inversely related to the SET percentage (Spearman’s rho = −0.36; *p* = 0.0002). High levels of the isoform were strongly indicative of a classic phenotype with an infiltrative or papillary pattern (*p* = 0.003) (Figure 3B).

### 3.4. AKT Isoforms Are Related to BRCA1 Expression and Homologous Recombination (HR) Status

**Figure 4:**
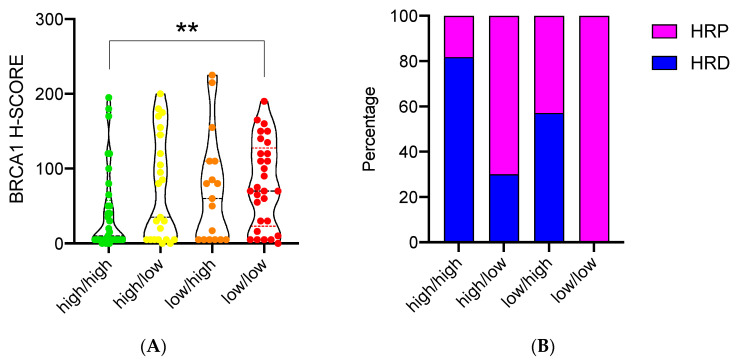
Association of AKT isoform categories with BRCA1 IHC expression and homologous recombination system (HR) status. (**A**) Violin plots representing the expression of BRCA1 (measured by the H-score method) among the four AKT categories; (**B**) bar chart showing the distribution of cases with proficient (HRP) and deficient (HRD) homologous recombination system among the four AKT categories. ** indicates statistical significance.

The four AKT categories were then associated with BRCA1 IHC expression as obtained by means of the H-score method. The results showed a trend towards an increase in BRCA1 expression moving from patients with a “high/high” pattern (H-score = 10) to those with a “low/low” pattern (H-score = 70). The difference between the two groups was statistically significant (*p* = 0.005) (Figure 4A). Accordingly, the expression of both AKT2 and AKT3 isoforms was strongly correlated with BRCA1 levels (rho = 0.27; *p* = 0.006 and rho = 0.20 *p* = 0.04, respectively).

The association between AKT groups and tumor BRCAness was partially supported by the results on the BRCA1/BRCA2 genetic testing, which was available for 36 patients. The distribution of HR-proficient and deficient cases, based on the BRCA1 and BRCA2 genetic testing, was significantly different between the four AKT categories (*p* = 0.003) as depicted in Figure 4B. HR deficiency was observed in 9 out of 11 patients (82%) of the “high/high” group (prevalent AKT1 expression), but in none (0%) of the 8 patients of the “low/low” group (prevalent AKT2 and AKT3 expression). Furthermore, higher rates of HR proficiency were also detected in the “high/low” class (7 out 10 patients), but not in the “low/high” one (3 out of 7 patients).

**Figure A2:**
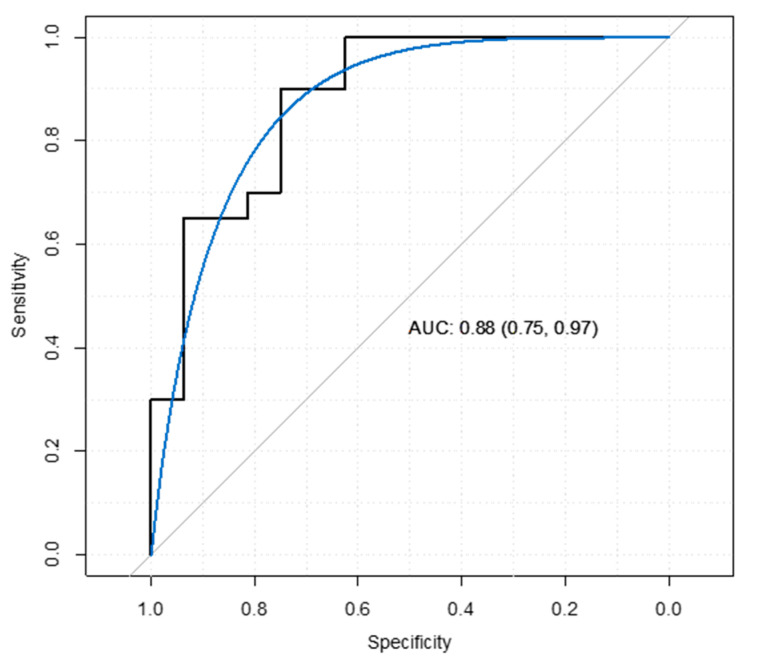
Validation of the logistic regression model by means of ROC curve and AUC value (AUC = 0.88).

Lastly, AKT1/AKT2, AKT1/AKT3, SET% and BRCA1 H-score were tested for their ability to predict the HR status by means of a logistic regression analysis. The results showed that AKT1/AKT3 and SET% were independent predictors of the HR status, as defined by the BRCA1 and BRCA2 genetic test (see Appendix A Table A3). A further receiver operating characteristic (ROC) analysis revealed that our model could accurately classify HRP and HRD cases with a good sensitivity and specificity (AUC = 0.88) (see Appendix A, Figure A2).

### 3.5. Data Validation

**Figure A3:**
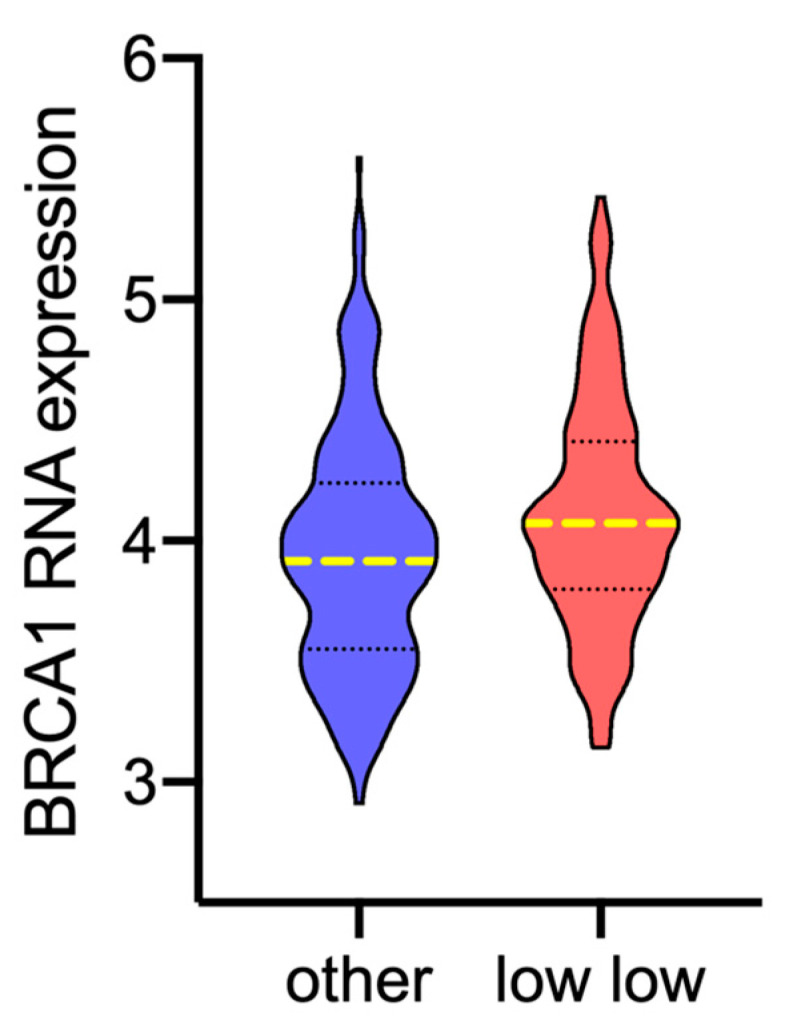
BRCA1 mRNA expression by Affymetrix microarray analysis (TCGA Firehose Legacy) in ”low/low” group compared to the other AKT categories (*p* = 0.0009).

Results from the TCGA dataset (*n* = 407 patients) confirmed a possible association between BRCA1 mRNA expression and the low/low AKT category, as BRCA1 expression was significantly higher in this group compared to the other AKT classes (*p* = 0.0009; Appendix A, Figure A3). Moreover, the expression of both AKT2 and AKT3 isoforms was significantly correlated with BRCA1 (rho = 0.37; *p* < 0.0001 and rho = 0.30 *p* < 0.0001, respectively).

## 4. Discussion

In the present study, we analyzed the mRNA expression of AKT isoforms in a cohort of 103 patients diagnosed with HGSOC. In agreement with Linnerth and colleagues [22], we found that AKT1 was the main isoform-sustaining AKT pathway as its expression prevailed on AKT2 and AKT3 in almost 70% of cases. However, the survival analysis clearly pointed out that AKT1 expression alone was not related to patients’ outcome. Possibly, other underlying mechanisms such as the reciprocal expression with the other two isoforms could be involved.

By calculating AKT1/AKT2 and AKT1/AKT3 ratios, we identified four AKT categories that resulted in being independent prognostic factors for patients’ survival. Furthermore, those categories were also associated with HGSOC morphological and immunophenotypical features. Women with tumors in the “low/low” group, where AKT2 and AKT3 expression prevailed over AKT1, had worse outcomes compared to the other categories. Their tumors frequently had a classic phenotype (mostly with an infiltrative and papillary pattern), psammoma bodies, a low mitotic rate and HR proficiency. These factors have already been associated with a poor outcome and response to chemotherapy [17] and, accordingly, in our study, the “low/low “group also displayed the highest rate of resistance.

On the other hand, patients of the “high/high” group, with a prevalent AKT1 expression over the other two isoforms, had the highest survival rates and preferably presented features associated with a more favorable outcome such as a SET morphology, absence of psammoma bodies, a higher number of mitoses and HR deficiency. In agreement with our findings, other authors reported that high levels of the AKT1 isoform could be related with the loss of BRCA1 expression and HR inhibition in breast cancer models, suggesting that a similar mechanism could be possible in HGSOC [23,24]. Furthermore, AKT1 has been identified as an age-associated gene in different non-human species, thanks to its ability to stimulate the insulin/IGF1 pathway and inhibit DNA repair mechanisms [25,26].

Patients included in the other two AKT categories, “high/low” and “low/high”, instead, had an intermediate outcome and were heterogeneous both histologically and immunophenotypically. Comparing those intermediate groups, the “high/low” with AKT1 prevailing on AKT2, but not AKT3, had more frequently classic features, lower mitoses and the presence of psammoma bodies, but, unexpectedly, it had a higher rate of response to chemotherapy and similar survival rates. This apparent discrepancy could be in part due to the partial completeness of clinical data, but it could also be hypothesized that more aggressive HGSOC phenotypes require the prevalence of both AKT2 and AKT3 over AKT1.

Taking our results where an association between AKT categories and patient survival was detected, it is possible that patients with a prevalent AKT1 expression (“high/high” group) may respond well to platinum-based chemotherapy and, thus, they could reach better outcomes. We could also hypothesize that the different expressions of AKTs could represent a potential predictive biomarker for a combinatorial treatment with PARP and AKT1 inhibitors, but we acknowledge that functional studies are needed to confirm our hypothesis and to prove that mRNA expression levels of AKTs isoforms correlate with their protein expression and activation. These results, if confirmed, could have important implications in the management of HGSOC. In an early phase clinical trial, Capivasertib, a potent and selective ATP competitive inhibitor of all three isoforms of AKT, demonstrated to be safe and active in *BRCA 1–2*-proficient or deficient tumors when combined with olaparib (a PARP inhibitor) [8]. Even the association of olaparib and alpelisib, an alpha-selective inhibitor of PI3K, has resulted in promising results specifically in patients with HGSOC, supporting the synergism between the inhibition of both pathways [27]. Nevertheless, in future phase II–III clinical trials, a tailored approach is needed, and potential predictive biomarkers of clinical benefit are not yet available.

On the other hand, patients in the “low/low” group seemed to be more resistant to first-line agents, at least in our cohort. If mRNA expression of AKT2 and AKT3 in the low/low group mirrors their protein levels, those patients could benefit from the combinatorial treatment with AKT2/AKT3 selective inhibitors. Similar considerations could be determined for the intermediate categories (“high/low” and “low/high”), where an association of the AKT1 inhibitor with an AKT2 or AKT3 inhibitor according to the prevalent isoform could represent a possible choice based on the mRNA results. As previously mentioned, in these groups, the response to first-line agents is more heterogeneous, although tumors included variables indicative of a poor outcome and resistance (classic features, low mitoses and psammoma bodies) more frequently in the “high/low” group, namely, the one where AKT3 prevailed over AKT1.

In this study AKT3 was notably implicated in determining the tumor phenotype and aggressiveness, although it was the prevalent isoform in a small subset of patients (3%). In agreement with our results, Li and colleagues found AKT3 upregulated in a chemotherapy-resistant group of patient-derived xenograft models of ovarian cancer [28]. In addition, other authors identified AKT3 as a key factor inducing tumor growth and invasiveness in ovarian and other neoplasms, including triple-negative breast cancer, which highly resembles the features of HGSOC [29,30]. Cristiano and co-workers also reported a specific role for AKT3 in the genesis of a subset of ovarian cancer by promoting the G2-M phase transition [31], but, contrary to their results, we found AKT3 expression inversely related with the mitotic rate (Spearman’s rho = −0.27; *p* = 0.006), suggesting its potential role in determining a tumor phenotype with “stemness-like” features, resistant to chemotherapy. This hypothesis was also supported by the positive correlation between AKT3 and BRCA1 IHC expression, a biomarker which has already been associated with stemness-like features and resistance in ovarian cancer [32].

Another emerging point of our results was the relationship between the tumor morphology, AKT isoforms and HR status. Considering the incompleteness of the HR status by BRCA1/BRCA2 genetic testing [33] as well as the expensiveness of current multigene panels for HRD testing, the prediction of homologous recombination proficiency/deficiency by means of AKT ratios, SET and BRCA1 expression could be of particular interest as a possible surrogate. Despite the small sample size, our logistic regression analysis suggested that the simultaneous assessment of the abovementioned features could be useful at least in discriminating tumors with somatic pathologic mutations on BRCA1 or BRCA2 genes, also resulting in a good level of sensitivity and specificity (0.75; 0.97 − AUC = 0.88). In our model, the BRCA1 H-score and AKT1/AKT2 ratios were not good predictors of the HR status (*p* = 0.3 and 0.14); however, validation by a ROC curve analysis excluding those variables resulted in lower AUC values (AUC = 0.86). In connection therewith, further studies are needed to seek whether AKT ratios, SET and BRCA1 expression can act as surrogates to predict the HRD status.

We acknowledge as a limit in this study the relatively small sample size and that the cohort of HGSOC spanned a broad period, where several surgical and therapeutical improvements were achieved. Nevertheless, no patient was treated with PARP inhibitors and AKT categories resulted to be independent prognostic factors even after the correction of the multivariate Cox regression model for the bevacizumab treatment (data not shown). We acknowledge that, to support the clinical utility of our results, they need to be validated in a broader cohort in a multisampling fashion to consider intratumor heterogeneity. Furthermore, the functionality of the AKTs isoforms and their causality in driving a specific HGSOC phenotype should be verified to support our hypotheses.

## 5. Conclusions

In conclusion, using a retrospective cohort of archive tissues, we identified four AKT-related survival categories linked to HGSOC histological and molecular features that could help in the possible selection of patients who could benefit from the combinatorial therapy with AKT and PARP inhibitors.

## Figures and Tables

**Table 1:** Clinicopathologic features of the cohort.

Features	N = 103, *n* (%)
Age at diagnosis mean	62 (range 30–79)
FIGO stage	
III	68 (68)
IV	32 (32)
NA	3
Progression after first-line CT	
No	24 (26)
Yes	70 (74)
NA	9
Residual tumor after surgery	
No	34 (36)
Yes	61 (64)
NA	8
Primary platinum response	
Never progressed	24 (27)
Resistant	25 (28)
Sensitive	39 (44)
NA	15
Anatomical sites	
Ovaries	85 (83)
Implants	18 (17)
BMI categories	
Normal	50 (56)
Obese	10 (11)
Overweight	19 (21)
Underweight	10(11)
NA	14
Bevacizumab	
No	64 (71)
Yes	26 (29)
NA	13
Survival status	
Alive	42 (41)
Dead	60 (59)
NA	1

CT—chemotherapy; BMI—body mass index; NA—not available.

**Table 2:** Prognostic factors for OS identified by multivariate Cox regression analysis.

Overall Survival (*N* = 93)			
	HR	95% CI	*P*-Value
Age at diagnosis	1.03	1.00–1.06	0.07
FIGO stage (III/IV)	2.65	1.50–4.68	0.0008 *
Residual tumor after surgery	0.81	0.47–1.41	0.5
AKT categories			
*high/high* ^1^	1		
*high/low*	2.43	1.13–5.19	0.02 *
*low/high*	2.01	0.87–4.68	0.1
*low/low*	3.46	1.71–7.03	0.0006 *
*Ph Test: p = 0.3*			

^1^ High/high group, namely, AKT1 prevalence, was taken as reference. * indicates statistical significance

**Table 3:** Clinicopathological features and their associations with AKT isoform categories.

	AKT Isoform Categories				
Clinicopathological Variables	High/High (33)	High/Low (23)	Low/High (17)	Low/Low (30)	*p*-Value
Tumor pattern INF MP PA PSE SD TR					
	5 (15)	9 (39)	0 (0)	6(20)	
	0 (0)	2 (9)	0 (0)	0 (0)	
	7 (21)	6 (26)	6 (35)	12 (40)	0.01 *
	7 (21)	4 (17)	8 (47)	5 (17)	
	9 (28)	2 (9)	2 (12)	5 (17)	
	5 (15)	0 (0)	1 (6)	2 (7)	
SET/Classic Classic SET					
	11 (33)	15 (65)	4 (23)	16 (53)	0.02 *
	22 (67)	8 (35)	13 (77)	14 (47)	
Mitoses (x10 HPF) Median (range)					
	23 (10–110)	16 (5–42)	22 (5–62)	17 (5–92)	0.03 *
Psammoma bodies Absent Present					
	31 (94)	17 (74)	17 (100)	23 (77)	0.03 *
	2 (6)	6 (26)	0 (0)	7 (23)	
N° lymphocytes (x 1HPF)					
Median (range)	10 (5–60)	10 (0–70)	10 (5–80)	20 (0–60)	0.5
Age at diagnosis					
Median (range)	65 (30–79)	64 (32–73)	61 (38–74)	65 (48–79)	0.5
FIGO stage					
III	22 (67)	17 (74)	12 (71)	18 (67)	0.9
IV	11 (33)	6 (26)	5 (29)	9 (33)	
NA	0	0	0	3	
Residual tumor					
No	11 (34)	7 (33)	6 (35)	10 (40)	1
Yes	21 (66)	14 (67)	11 (65)	15 (60)	
NA	1	2	0	5	
Primary platinum response					
Never progressed	8 (29)	4 (22)	6 (37)	6 (23)	
Resistant	6 (21)	2 (11)	4 (25)	13 (50)	0.07
Sensitive	14 (50)	12 (67)	6 (38)	7 (27)	
NA	5	5	1	4	

INF—infiltrative; MP—micropapillary; PA—papillary; PSE—pseudo-endometrioid; SD—solid; TR—transitional-like; NA—not available; * indicates statistical significance.

## Data Availability

Data supporting reported results are available on request to the corresponding authors.

## Appendix A

**Table A1:** Annealing temperature and oligonucleotide sequences used for AKT1 gene amplification on droplet digital PCR.

Gene	Primers and Probe	Length	Annealing Temp.
AKT1	Forward 5′- CCA CTG TCA TCG AACGCA CCT - 3′	77 bp	56 °C
	Reverse 5′ - CAC AGT CTG GAT GGC GGT TGT - 3′		
	Probe 5′ - CTG AGG AGC GGG AGG AG - 3′		

**Table A2:** Prognostic factors for PFS identified by multivariate Cox regression analysis.

Progression-Free Survival (*N* = 62)			
	HR	95% CI	*p*-Value
Age at diagnosis	1.02	1.00–1.05	0.1
FIGO stage (III/IV)	1.14	0.63–2.03	0.7
Residual tumor after surgery	2.00	1.11–3.61	0.02 *
AKT categories			
*High/high* ^1^	1		
*High/low*	1.21	0.57–2.57	0.6
*Low/high*	1.37	0.61–3.07	0.4
*Low/low*	1.90	0.95–3.79	0.07
*Ph Test: p = 0.5*			

^1^ High/high group, namely, AKT1 prevalence, was taken as reference. * indicates statistical significance.

**Table A3:** Estimates of the binomial logistic regression model for variables influencing HR status.

Logistic Regression Model				
	Estimate	Std. Error	z-Value	*p*-Value
Intercept	5.42	2.08	2.61	0.009 *
AKT1/AKT2	−0.58	0.39	−1.48	0.14
AKT1/AKT3	−0.30	0.15	−1.98	0.04 *
SET%	−0.02	0.01	−2.00	0.04 *
BRCA1 H-score	−0.01	0.01	−1.05	0.3
*Hosmer and Lemeshow test: Chi-square = 11.51; df = 8; p = 0.2*				

* indicates statistical significance.
